# Characterization and metabolic synthetic lethal testing in a new model of SDH-loss familial pheochromocytoma and paraganglioma

**DOI:** 10.18632/oncotarget.23639

**Published:** 2017-12-22

**Authors:** John Smestad, Oksana Hamidi, Lin Wang, Molly Nelson Holte, Fatimah Al Khazal, Luke Erber, Yue Chen, L. James Maher

**Affiliations:** ^1^ Mayo Clinic Medical Scientist Training Program, Mayo Clinic College of Medicine and Science, Rochester, MN, USA; ^2^ Department of Biochemistry and Molecular Biology, Mayo Clinic, Rochester, MN, USA; ^3^ Division of Endocrinology, Diabetes, and Nutrition, Mayo Clinic, Rochester, MN, USA; ^4^ Department of Pediatric and Adolescent Medicine, Mayo Clinic College of Medicine and Science, Rochester, MN, USA; ^5^ Department of Biochemistry, Molecular Biology and Biophysics, University of Minnesota at Twin Cities, Minneapolis, MN, USA

**Keywords:** transcriptomics, epigenomics, proteomics, synthetic lethality, lactate dehydrogenase

## Abstract

Succinate dehydrogenase (SDH)-loss pheochromocytoma and paraganglioma (PPGL) are tumors driven by metabolic derangement. SDH loss leads to accumulation of intracellular succinate, which competitively inhibits dioxygenase enzymes, causing activation of pseudohypoxic signaling and hypermethylation of histones and DNA. The mechanisms by which these alterations lead to tumorigenesis are unclear, however. In an effort to fundamentally understand how SDH loss reprograms cell biology, we developed an immortalized mouse embryonic fibroblast cell line with conditional disruption of *Sdhc* and characterize the kinetics of *Sdhc* gene rearrangement, SDHC protein loss, succinate accumulation, and the resultant hypoproliferative phenotype. We further perform global transcriptomic, epigenomic, and proteomic characterization of changes resulting from SDHC loss, identifying specific perturbations at each biological level. We compare the observed patterns of epigenomic derangement to another previously-described immortalized mouse chromaffin cell model of SDHB loss, and compare both models to human SDH-loss tumors. Finally, we perform analysis of SDHC synthetic lethality with lactate dehydrogenase A (LDHA) and pyruvate carboxylase (PCX), which are important for regeneration of NAD+ and aspartate biosynthesis, respectively. Our data show that SDH-loss cells are selectively vulnerable to LDH genetic knock-down or chemical inhibition, suggesting that LDH inhibition may be an effective therapeutic strategy for SDH-loss PPGL.

## INTRODUCTION

Pheochromocytoma and paraganglioma (PPGL) are rare neuroendocrine tumors arising from chromaffin cells of the adrenal medulla and autonomic sympathetic and parasympathetic paraganglia, respectively. Each year there are approximately 500 to 1600 new PPGL cases in the United States, with the combined estimated annual incidence of ∼0.8 per 100,000 person-years [1]. More than 30% of PPGL are hereditary with greater than 40% penetrance depending on genotype and up to 50% develop metastases in certain hereditary germline mutations [2, 3].

Mutations in tumor-suppressing genes encoding subunits of the succinate dehydrogenase (SDH) complex (SDHA, SDHB, SDHC, and SDHD, i.e. SDHx genes) and the required assembly factor that flavinates SDHA (SDHAF2) can inhibit SDH activity and thereby cause hereditary and (apparently) sporadic PPGL [4–9]. Syndromic PPGL is also seen as a part of multiple endocrine neoplasia type 2 (MEN2), von Hippel-Lindau (VHL) disease, and neurofibromatosis type 1 (NF1). Additionally, novel mutations associated with PPGL continue to be discovered. These include mutations in transmembrane protein 127 (TMEM127), myc-associated factor X (MAX) genes, somatic gain-of-function mutations in the gene encoding hypoxia-inducible factor 2α (HIF2A), and pathogenic germline mutations in the FH gene encoding fumarate hydratase [10–13].

Decreased activity of SDH due to mutations in SDHx genes leads to increased intracellular concentrations of succinate, a tricarboxylic acid (TCA) cycle intermediate thought to be a crucial tumorigenic oncometabolite SDH-deficient PPGL [6, 8, 14–17]. Succinate is a 2-ketoglutarate analog, and is an inhibitor of an entire class of more than 40 dioxygenase enzymes that utilize dioxygen, iron, and 2-ketoglutarate in hydroxylation and demethylation reactions [18]. Inhibition of prolyl hydroxylases by succinate is believed to activate pseudohypoxic signaling, which may be tumorigenic [14, 15, 19, 20]. Additionally, succinate inhibition of TET DNA demethylases and Jumonji domain histone demethylases is believed to cause hypermethylation of histones and DNA, resulting in global transcriptional perturbation [21–24]. Despite these insights, however, it remains unclear how SDH loss and resultant pseudohypoxia and global epigenomic derangement are tumorigenic.

In an effort to fundamentally understand how SDH loss reprograms cell biology, we developed an immortalized mouse embryonic fibroblast cell line with conditional disruption of *Sdhc* and characterize the kinetics of *Sdhc* gene rearrangement, SDHC protein loss, succinate accumulation, and the resultant hypoproliferative phenotype. We study this new model of SDH loss by performing global transcriptomic, epigenomic, and proteomic characterization of changes resulting from SDHC loss, identifying specific perturbations at each biological level. We compare the observed patterns of epigenomic derangement to another previously described immortalized mouse chromaffin cell model of SDHB loss, and compare both models to human SDH-loss tumors.

In the absence of a fully functional TCA cycle due to SDH deficiency, cells rewire their metabolic network and become dependent on alternative pathways for proliferation and survival. Genetic and hypoxia-mediated disruptions of the TCA cycle have been suggested to result in greater reliance on glycolysis and/or reductive carboxylation of glutamine for the provision of carbon for anaerobic purposes [25–27]. Prior investigations suggest that LDHA, an enzyme that catalyzes the reduction of pyruvate to lactate for NAD+ regeneration, is critical for survival of SDH-deficient cells [28, 29]. In parallel, it has been suggested that PCX is an essential enzyme for aspartate biosynthesis, particularly in glycolytic cells that lack TCA cycle function [30]. In the current study, we characterize the sensitivity of our new SDH-loss model to genetic loss of LDHA and PCX via lentivirus-mediated shRNA knockdown. We additionally characterize the sensitivity of SDH loss cells to chemical inhibition of LDH. Our data show that SDH-loss cells are selectively vulnerable to LDH genetic knock-down or chemical inhibition, suggesting that LDH inhibition may be an effective therapeutic strategy for SDH-loss PPGL.

## RESULTS

### Genetic and phenotypic characterization of SDHC-loss iMEF model

**Figure 1:**
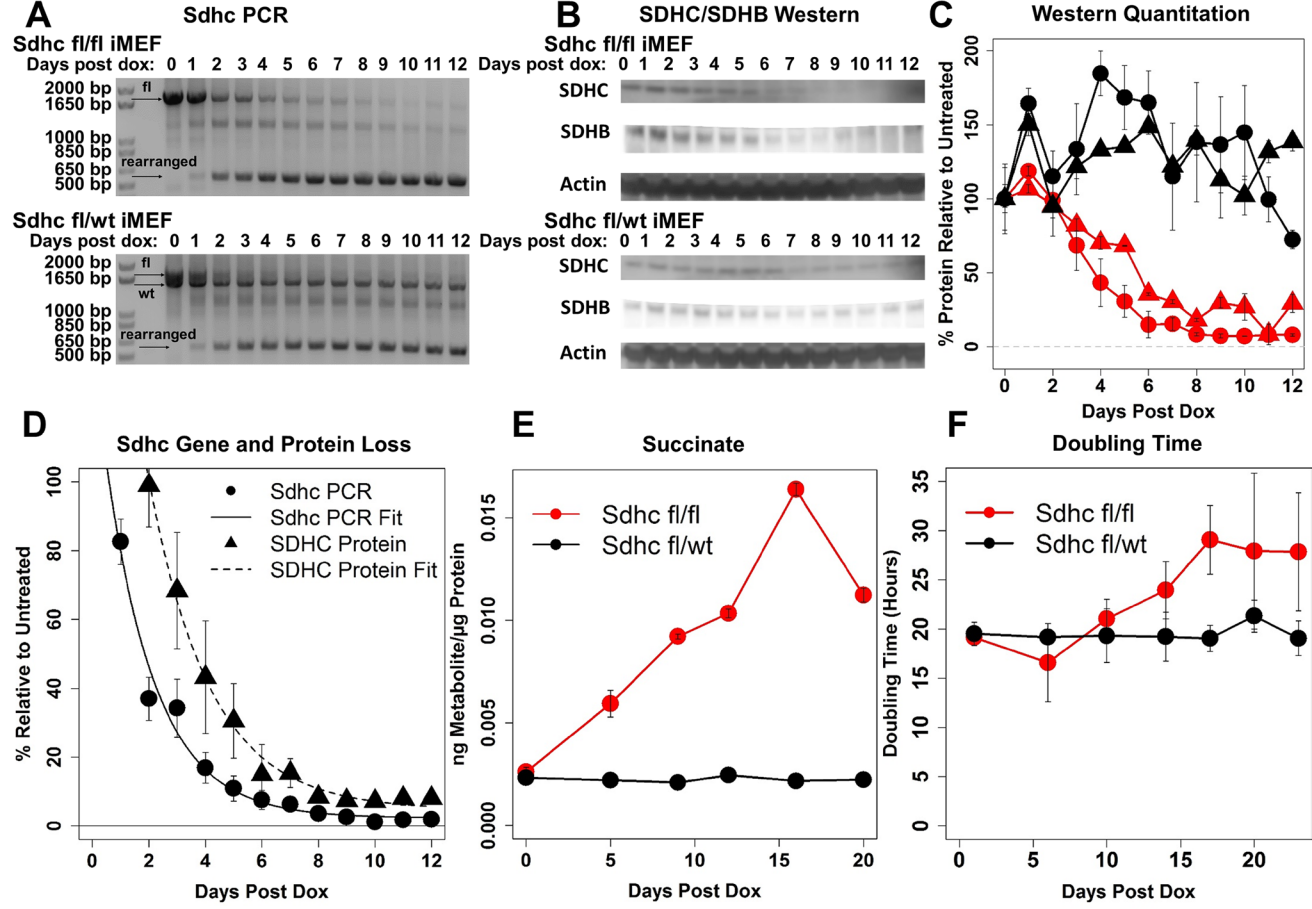
Genetic and phenotypic characterization of SDHC-loss iMEFs (**A**) PCR analysis of *Sdhc* gene rearrangement using primers flanking [floxed] exon 4, resulting in production of a shortened PCR product upon Cre-mediated gene rearrangement. (**B**) Western blot analysis of SDHC and SDHB protein loss following *Sdhc* gene rearrangement. (**C**) Western blot quantitation. Colors indicate respective iMEF line (red, experimental; black, control). Symbols correspond to quantified protein (circles: SDHC; triangles: SDHB). Welch two-sample *t*-test of SDHC protein amount at day 12 quantified for experimental and control lines yields *p*-value of 0.004 (*N* = 6 experimental replicates). Similar statistical analysis of SDHB protein amount yields *p*-value of 3E-5. (**D**) Exponential decay models of *Sdhc* gene rearrangement and protein loss. DNA rearrangement and SDHC protein half-lives are 1.76 and 2.17 d, respectively. Midpoints for DNA rearrangement and SDHC protein loss curves occur at 1.8 and 3.6 d, respectively. (**E**) Measured intracellular succinate abundance. Values are normalized to total protein. (**F**) Quantitation of cell population doubling time. Welch two-sample *t*-test of doubling time difference between experimental and control lines at day 22 yields a *p*-value of 0.004.

We developed experimental (*Sdhc* fl/fl) and control (*Sdhc* fl/wt) immortalized mouse embryonic fibroblast (iMEF) cell lines in which *Sdhc* gene rearrangement can be triggered by doxycycline induction of Cre recombinase expression. These iMEF lines were obtained from mouse embryos using animals by FLP recombinase manipulation of a *Sdhc* gene trap allele with exon 4 spanned by Cre recombinase recognition sequences, developed by the Wellcome Trust Sanger Institute. Both experimental and control iMEFs were treated with doxycycline and sampled over time to monitor *Sdhc* gene rearrangement using PCR primers flanking floxed exon 4 that reveal a shortened PCR product upon Cre-mediated gene rearrangement (Figure 1A). Following *Sdhc* gene rearrangement, loss of SDHC and SDHB proteins was verified by Western blot analysis (Figure 1B, 1C). Quantification of the *Sdhc* floxed allele and SDHC protein decay rates using exponential decay fitting revealed DNA rearrangement and SDHC protein half-lives were ∼1.8 and ∼2.2 d, respectively, with midpoints at 1.8 and 3.6 days (Figure 1D). Intracellular succinate levels were then profiled using GC/MS approaches. Succinate was found to be elevated in experimental cells following induction of SDHC loss, but not control cells (Figure 1E). We then characterized cell population doubling time over a time-course, revealing increased doubling times for SDH-loss cells (Figure 1F).

### Global transcriptional responses to SDHC loss

**Figure 2:**
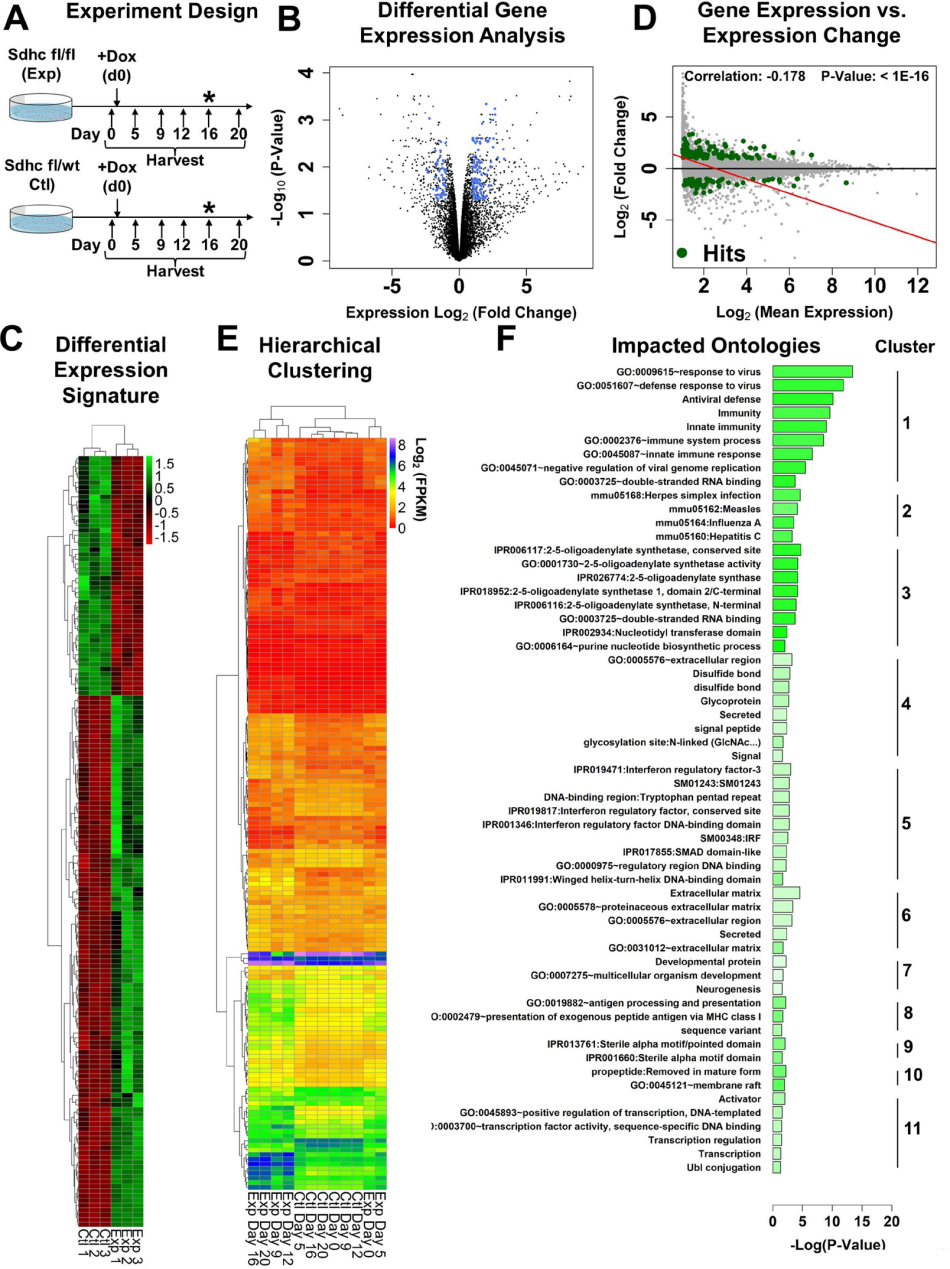
Transcriptomic characterization of SDHC-loss iMEFs (**A**) Schematic representation of Sdhc loss time-course design used for RNA-seq experiments. Both Sdhc fl/fl (experimental) and Sdhc fl/wt (control) iMEFs are treated with doxycycline and sampled over time to monitor transcriptional responses to Sdhc loss. Asterisks (*) indicate time point used for collection of biological replicate (*N* = 3) specimens used for differential gene expression analysis. (**B**) Volcano plot showing expression differences between experimental and control lines at day 16. Subset of genes identified as differentially expressed (log2FC > 1 and adjusted *p*-value < 0.05 and which showed less than 2-fold expression difference between the two lines at day 0; *N* = 161 genes) are highlighted in blue. (**C**) Heat map showing relative expression of identified differentially expressed genes between experimental and control cell lines and biological replicates. (**D**) Analysis of observed gene expression changes as a function of baseline expression value in control cells. Subset of differentially-expressed genes is highlighted in green. (**E**) Unsupervised hierarchical clustering of time-course experiment gene expression profiles for experimental and control iMEF lines using identified differentially-expressed genes. Experiment design is as depicted in A. Dendrogram structure at top of plot indicates degree of similarity between clustered gene expression signatures. (**F**) DAVID functional annotation enrichment analysis and clustering of impacted gene ontologies. Color indicates average gene expression change for impacted genes mapping to each ontology (green: up-regulation in experimental line; red: down regulation in experimental line).

We next characterized immediate transcriptome-wide changes in response to SDHC loss. For this purpose, we employed a time-course experimental design in which we triggered rearrangement of *Sdhc* floxed alleles in experimental and control iMEF lines and then iteratively sampled these cell cultures over time for RNA-seq profiling to monitor time-dependent changes in gene expression (Figure 2A). To identify differentially-expressed genes, we leveraged biological triplicate RNA-seq experiments collected at day 16 post induction to perform a differential gene expression analysis [absolute log_2_(fold-change) >1 and adjusted *p*-value < 0.05]. An unfiltered differential genes expression analysis identified 610 genes that were differentially expressed between experimental and control lines at day 16 (absolute log_2_(fold-change) >1 and adjusted *p*-value < 0.05, Supplementary Table 1). We further filtered the results to consider only genes whose expression was similar (within two-fold) between experimental and control lines at day 0 (Figure 2B, 2C). This filtering retained 161 genes (Supplementary Table 2). A majority (111; 69%) of these differentially-expressed genes were up-regulated in the experimental line relative to control.

Correlation of gene-specific expression change with baseline expression levels in the control cell line revealed a negative correlation (−0.178, *p*-value < 1E-16) between expression change and baseline expression value genome-wide (Figure 2D). Further examination of this correlation using a linear fit to the full dataset revealed that genes having a baseline expression value < 6 fragments per kilobase million (FPKM) tend to increase in expression in the context of SDHC-loss, while genes having expression >6 FPKM tend to decrease in expression. This finding that patterns of SDHC-loss transcriptional change correlate with baseline expression values in normal cells has not previously been reported, and points to distinct transcriptional activating and repressing effects that operate on these gene subsets in the context of SDHC-loss.

We next asked whether the expression levels of the 161 genes differentially-expressed at day 16 after SDH loss are adequate to classify experimental and control time-course samples into distinct groups. We also wished to ascertain at what time major differences in gene expression begin to emerge. We therefore performed unsupervised hierarchical clustering of gene expression profiles using the identified differentially-expressed genes. This analysis (Figure 2E) reveals that experimental time-course day 0 and 5 samples have high similarity to the control samples, and that major changes in gene expression begin to emerge by day 9, and become more pronounced at later time points. This is consistent with a model in which transcriptional differences between the two iMEF lines emerge after induction of *Sdhc* gene rearrangement, with dramatic differences between day 0–5 and day 9–20 experimental iMEF line transcriptional profiles, and corresponds with the observed delay in loss of previously-expressed SDHC protein.

To gain insight into functional implications of SDH loss in the fibroblast context, we performed functional annotation enrichment analysis and term clustering on the list of identified differentially-expressed genes using the DAVID functional annotation database [31]. This analysis (Figure 2F, Supplementary Table 3) revealed several clusters of terms describing the fibroblast response to SDHC loss. The most highly enriched term sets (clusters #1, #2, and #3) reveal significant induction of genes involved in innate immunity and antiviral response. Other prominent patterns in the enriched term sets include induction of genes involved in disulfide bond formation (cluster #4), interferon genes (cluster #5), and extracellular matrix genes (cluster #6).

### Genome-wide DNA methylation patterns in response to SDHC loss

**Figure 3:**
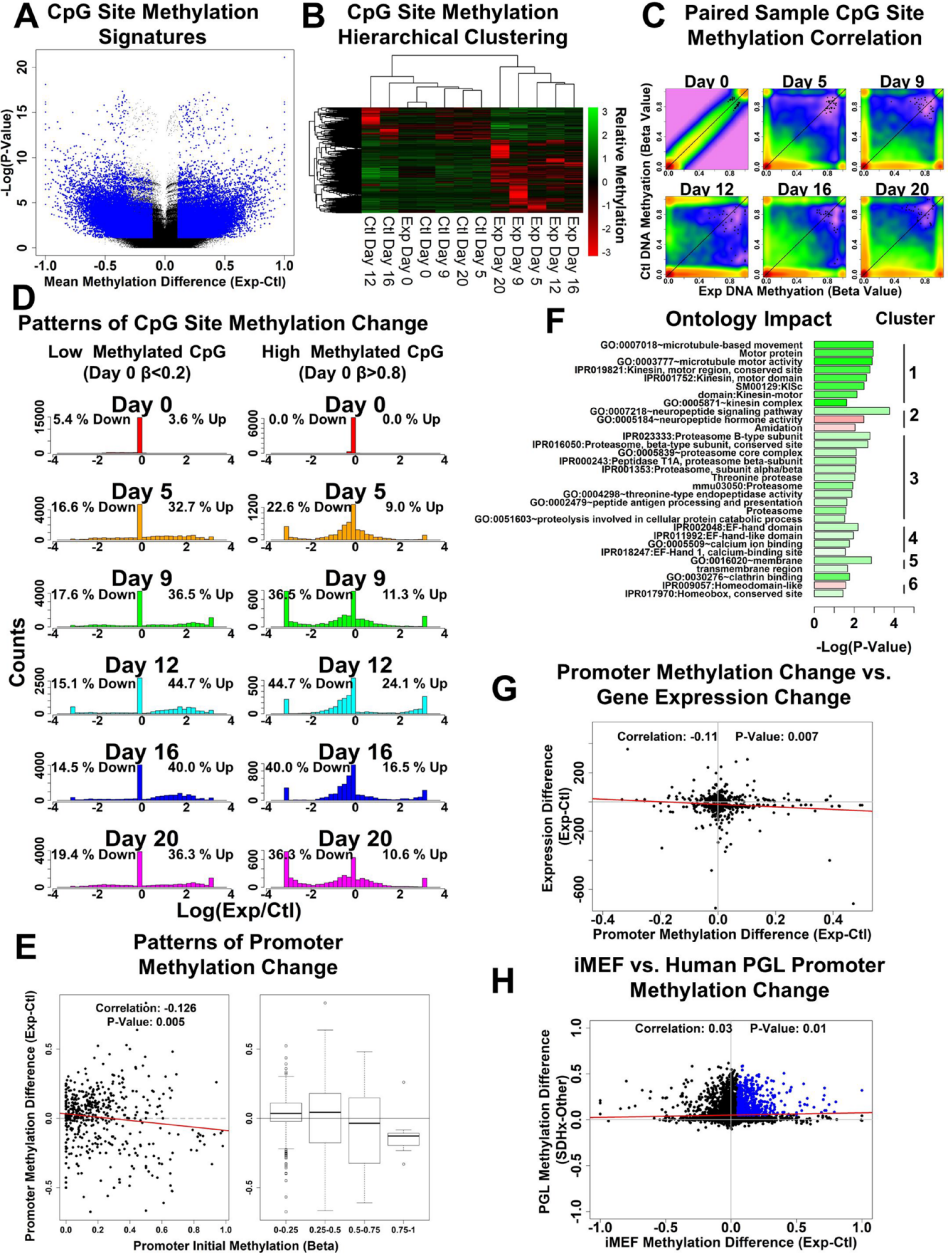
Analysis of genome-wide methylation patterns in SDHC-loss iMEFs (**A**) Volcano plot showing CpG site mean methylation difference versus –log(*p*-value) from RnBeads differential methylation analysis. Comparison was generated between control time series specimens and experimental specimens, excluding day 0. Blue dots correspond to the top 0.1 quantile of the dataset, as quantified by methylation combined rank. (**B**) Unsupervised hierarchical clustering of samples based upon CpG site DNA methylation patterns. The data used for clustering includes the top 0.1 quantile of CpG sites, ordered by methylation combined rank. (**C**) Correlation heat maps showing the emergence of CpG site DNA methylation difference between experimental and control iMEF lines following induction of SDHC loss with doxycycline. Only differences emerging after day 0 are shown. Colors correspond to data point density (red: high; green: intermediate; blue: low). (**D**) Time course analysis of CpG site methylation change, separated according to day 0 methylation status. Left histograms illustrate change in DNA methylation for subset of CpG sites with little initial methylation (day 0 beta value < 0.2). Right histograms illustrate change in DNA methylation for subset of CpG sites with high initial methylation (day 0 beta value > 0.8). Quantitations overlaid on plots indicate the percentage of data CpG sites having a 10-fold increase or decrease in methylation between experimental and control lines. (**E**) Patterns of promoter methylation change shown as a function of initial (day 0) methylation beta value. (**F**) Analysis of gene ontologies impacted by differential methylation of gene promoters. Dataset used for gene ontology searching includes the top 0.1 quantile of data, ranked according to promoter differential methylation combined rank. Color scale indicates mean degree of methylation change for promoters of genes identified as impacted and mapping to that given ontology (green: hypermethylated; red: hypomethylated). (**G**) Integrative analysis of promoter methylation change and gene expression change at day 20 post doxycycline initiation. Promoter methylation change is calculated as the difference between experimental and control cell line promoter beta values. Gene expression change is calculated as the difference between experimental and control line gene expression values (FPKM). Data included in this analysis are the top 0.05 quantile of data ranked according to adjusted *p*-value from differential gene expression analysis. (**H**) Comparison of iMEF SDHC-loss and PPGL SDHx-loss promoter differential methylation patterns. Methylation difference in iMEFs is calculated as a difference of day 20 promoter beta values for experimental minus control lines. Methylation difference for human PPGL tumors is calculated as the difference of beta values for SDHx tumors minus all other PPGL tumors, using the nearest CpG site to a given gene promoter as a surrogate for promoter methylation beta value.

To extend our analysis of SDH loss to epigenetic effects, we performed global profiling of DNA methylation patterns. As in gene expression analysis, we adopted a time-course experimental design in which we monitor differences in DNA methylation that emerge between experimental and control iMEF lines upon induction of SDHC loss with doxycycline. For each time point, isolated genomic DNA from experimental and control cell lines was profiled by reduced representation bisulfite sequencing (RRBS) [32] to map genome-wide patterns of DNA methylation. Using functionality of the RnBeads R package, we performed a differential DNA methylation analysis to identify CpG site methylation differences between experimental and control sample series, excluding the day 0 experimental sample, with the goal of identifying changes that emerge between the iMEF lines after day 0. The results of this differential methylation analysis (Figure 3A) display the calculated DNA methylation beta value difference for individual CpG sites on the x-axis and the significance of the statistical comparison on the y-axis, highlighting the top 0.1 quantile of the dataset in blue as quantified by DNA methylation combined rank. We also performed this analysis on CpG sites mapping to annotated CpG islands and promoters (Supplementary Figure 1A and 1B, respectively).

The top 0.1 quantile of differentially-methylated CpG sites were then used to perform unsupervised hierarchical clustering of time-course samples following induction of SDHC loss (Figure 3B). Impressively, the methylation information at these loci was sufficient to produce a relational dendrogram that reveals high similarity between experimental day 5–20 specimens and similarly clusters all control specimens together. This CpG site methylation-based clustering also indicated a high degree of similarity between the control specimens and the experimental day 0 specimen, as expected. Clustering analyses were also performed for CpG sites mapping to annotated CpG islands and promoters (Supplementary Figure 1C and 1D, respectively). All three analyses support the conclusion that meaningful differences in methylation between iMEF lines emerge after day 0 and are attributable to SDHC loss in the experimental line.

We then examined specific patterns of DNA methylation change at CpG sites that emerge between experimental and control iMEF lines following induction of SDHC loss. For this analysis, only CpG sites in the top 0.1 quantile of differential expression combined rank, and which did not have significant difference at day 0 (beta value difference < 0.2) were considered. Shown in Figure 3C, and seen most prominently at days 12 through 20, dramatic differences emerge in CpG site methylation. In particular, CpG sites that have either a very low or very high beta value in the control line tend to display an off-diagonal gradient of beta values between 0 and 1 in the experimental line. A similar phenomenon was observed to a lesser extent when performing the analysis on CpG sites mapping to annotated CpG islands and promoters (Supplementary Figure 1E and 1F, respectively).

Since the majority of CpG sites in the control cell line appear to have either a very low or very high beta value, we then asked how these two CpG site populations change in methylation following induction of SDHC loss. We filtered the dataset to keep only CpG sites that have low (beta value < 0.2) or high (beta value > 0.8) methylation at day 0 in both experimental and control lines and analyzed the patterns of methylation change separately for these respective CpG site lists (Figure 3D). For CpG sites with a low day 0 beta value, the dominant pattern of CpG site methylation change after induction of SDHC loss was found to be hypermethylation, with 44% of CpG sites hypermethylated in the experimental line at day 12, and only 15% of sites hypomethylated. This result is consistent with the current paradigm of succinate-mediated inhibition of Tet demethylases, resulting in global hypermethylation of DNA [33]. We estimate that this pattern of hypermethylation of CpG sites having initially low methylation beta values affects approximately 13% of CpG sites genome wide. For CpG sites with a high day 0 beta value, however, the pattern was opposite. Among these CpG sites, 24% were seen to be hypermethylated in the experimental line at day 12, whereas 44% were seen to be hypomethylated. This finding that highly methylated CpG sites tend to become hypomethylated in the context of SDHC loss is novel and challenges the simple paradigm of global hypermethylation due to Tet enzyme inhibition and suggests a genome-wide averaging of DNA methylation. We estimate that this pattern of hypomethylation of CpG sites having initially high methylation beta values affects approximately 6% of CpG sites genome wide.

We then extended this DNA methylation analysis by studying aggregate promoter methylation patterns and how initial (day 0) methylation state correlated with methylation difference between experimental and control lines at day 20. Interestingly, we identified a statistically-meaningful negative correlation (−0.126, *p*-value = 0.005) between initial promoter methylation status and observed day 20 methylation difference (experimental minus control beta values). This result further supports the conclusion that initial methylation status is correlated with observed methylation changes (Figure 3E).

We next asked whether the gene promoters most impacted by differential methylation preferentially impact specific gene ontologies. We extracted the gene identifiers for the top 0.1 quantile of differentially-methylated promoters, as quantified by combined methylation rank, and used this list to perform functional annotation enrichment analysis and term clustering using the DAVID functional annotation database [31]. This analysis revealed several clusters of terms found to be preferentially impacted (Figure 3F, Supplementary Table 4). Notably, top impacted functional categories include microtubule-related components (cluster #1), neuropeptide signaling pathway (cluster #2), and proteasome components (cluster #3). These ontologies were not among the top impacted ontologies at the transcriptome level, suggesting that the most meaningful changes in methylation do not necessarily correspond to the most meaningful transcriptomic changes.

We assessed whether there is a correlation between observed changes in gene expression, quantified via RNA-seq, and observed changes in promoter DNA methylation, quantified via RRBS. We searched for a statistically-significant correlation between expression difference (experimental minus control FPKM values) and promoter DNA methylation difference (experimental minus control beta values). As shown in Figure 3G we found an intriguing negative and statistically-meaningful correlation (−0.11, *p*-value = 0.007) between the two variables, suggesting that increased promoter methylation may contribute to reduced gene expression. This is consistent with the current paradigm of promoter DNA hypermethylation being generally repressive of gene expression. We also identified day 0 promoter hypermethylation as correlating with increased day 20 experimental line gene expression (Supplementary Figure 1G). Furthermore, Supplementary Figure 1H shows that genes with increased expression in the experimental line tend to have a higher number of CpG sites per promoter (median = 23), relative to genes that do not change expression (median = 17), and that the opposite is true for down-regulated genes (median = 15). The basis for these phenomena are unclear.

Next, we assessed whether the observed *Sdhc*-dependent differences in promoter methylation patterns in our iMEF model correlate with SDHx-loss methylation patterns in these same genes in human PPGL tumors. Using publically-available datasets, we calculated the mean gene-specific promoter beta value differences for human SDHx-loss tumors relative to all other PPGL tumors. Strikingly, we observed a weak but statistically-significant correlation (0.03, *p*-value = 0.01) between the observed methylation differences in SDHC-loss iMEFs and SDHx-loss human PPGL tumors (Figure 3H). This indicates that some fundamental aspect of the observed epigenetic response to SDH loss may be conserved across species and cell types. We also assessed the degree of correlation between average promoter beta values averaged across all PGL tumors and day 0 iMEF promoter beta values. This analysis revealed a 33% correlation (Supplementary Figure 2A).

Prior to this report, another group reported the generation and epigenomic characterization of a spontaneously immortalized mouse chromaffin cell model system (imCC) for SDHB loss [33]. We therefore asked to what degree the epigenomic patterns observed in our iMEF model system are similar to this other model. Analysis of promoter methylation patterns in control iMEFs and imCCs revealed a surprisingly high correlation (87%) between these disparate cell types (Supplementary Figure 2B). We measured the similarity of differential promoter methylation patterns between experimental and control lines in these two cell types. This revealed a small but statistically significant correlation (0.051, *p*-value = 2E-10) between the observed epigenomic responses (Supplementary Figure 2C). We then asked how SDH loss patterns of promoter methylation difference between SDHC-loss iMEFs, SDHB-loss imCCs, and human SDHx-loss PPGL tumors compare. The results of this analysis (Supplementary Figure 2D) reveal that, although SDHC-loss iMEF methylation differences have statistically-significant correlation with SDHx-loss PPGL and SDHB-loss imCC methylation differences, the correlation between SDHx-loss PPGL and SDHB-loss imCC methylation differences is approximately 5-fold stronger. This result suggests that, with respect to gene-specific differences in the epigenome, the imCC model system may more closely approximate human PPGL tumors. This result is not surprising given the cell types involved.

We tested whether specific gene promoters are identified as hypermethylated in SDHC-loss iMEFs, SDHB-loss imCCs, and human SDHx-loss PPGL tumors. For this analysis, we considered only gene promoters with a beta difference value (experimental minus control) >0.05. A Venn diagram illustration of the hypermethylation dataset overlaps is presented in Supplementary Figure 2E. 282 genes were identified as hypermethylated in all three datasets, representing a conserved list of genes that become hypermethylated in SDH-loss context regardless of species and cell type. DAVID functional annotation enrichment analysis [31] and term clustering performed on this list identified several annotation term clusters (Supplementary Figure 3). Notably, several clusters were identified suggesting a conserved pattern of hypermethylation affecting ECM components and cell membrane (clusters #2, 13, 14), transcription (clusters #4, 16), Wnt signaling (clusters #8, 10), calcium signaling (cluster #12), homeobox factors (cluster #15), and collagen (cluster #17).

### Global proteomic changes in response to SDHC loss

**Figure 4:**
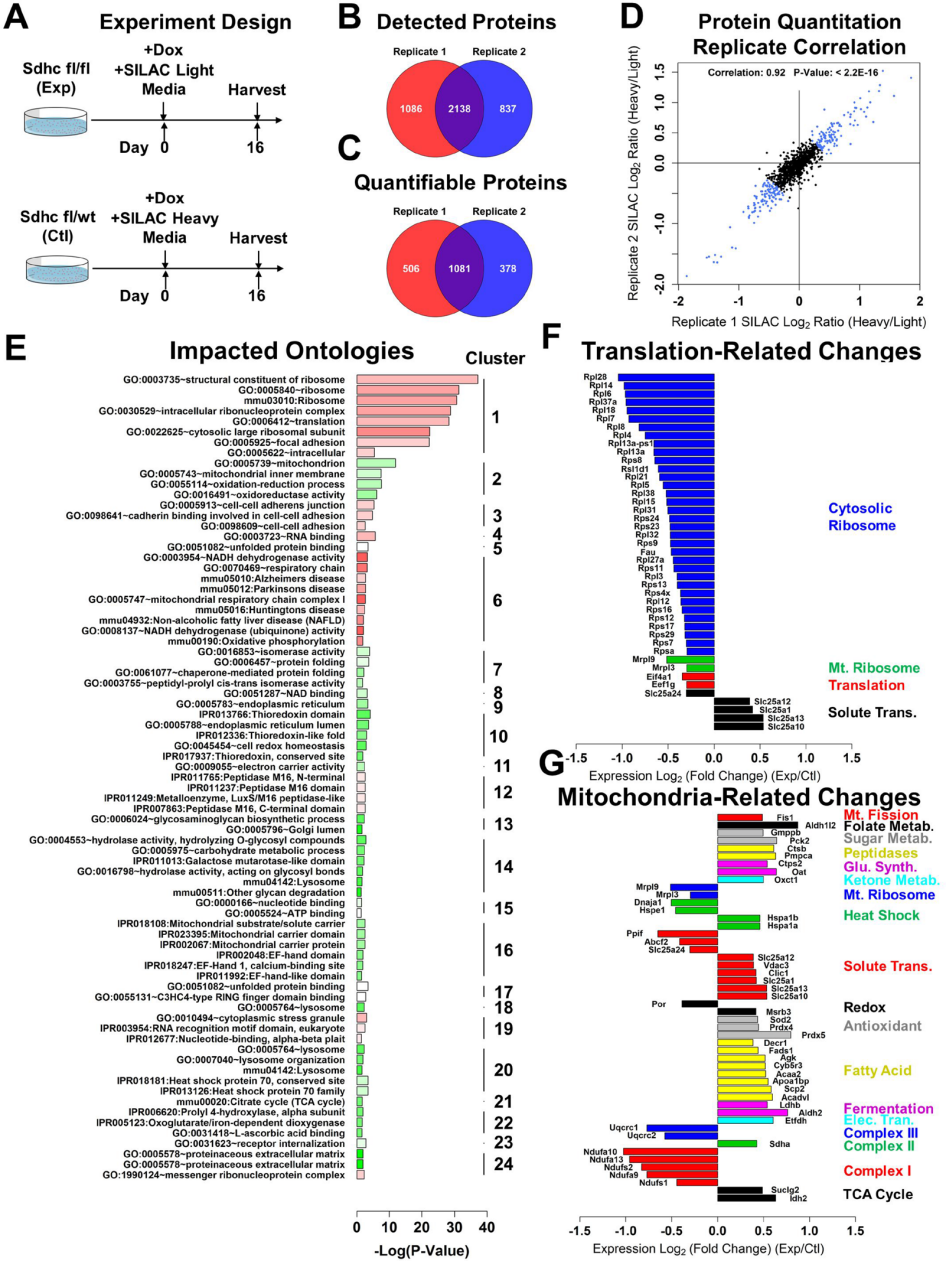
Global proteomic characterization of Sdhc loss iMEFs (**A**) SILAC experimental design. *Sdhc* fl/fl (experimental) and *Sdhc* fl/wt (control) cell lines were induced with doxycycline and grown in SILAC light and heavy medium, respectively. Heavy SILAC medium contained C13-labeled lysine and arginine. At day 16, cell pellets were harvested, pooled, proteolytically digested, and the ratios of heavy and light peptides quantified via mass spectrometry. (**B**) Venn diagram showing overlap of detected proteins identified in two biological replicate heavy/light labeling experiments. (**C**) Venn diagram showing overlap of quantifiable proteins in two biological replicate heavy/light labeling experiments. (**D**) Analysis of correlation between replicate protein quantitation experiments. Plot axes show calculated heavy/light log_2_-transormed ratios. Prior to log-transformation, data on each axis were independently normalized to median values. (**E**) Analysis of gene ontologies, KEGG pathways, and INTERPRO terms impacted at the protein level. Bar height corresponds to the degree of statistical significance in the function term enrichment analysis. Color of the bar corresponds to the magnitude and direction of protein abundance change in experimental vs. control iMEF cell lines (red: down-regulation in experimental line; green: up-regulation in experimental line). (**F**) Translation-related changes in protein expression. Shown are log_2_(fold-change) values (x-axis) for all quantified proteins with annotation mapping to translation-related cluster 1 (panel E). Positive values indicate increased expression in the experimental iMEF line relative to control. Quantified proteins are ordered according to specific cellular functions. (**G**) Mitochondria-related changes in protein expression. Display parameters are the same as in F.

We then assessed the global impact of SDHC loss upon relative protein abundance in experimental and control cell lines to see if observed transcriptomic changes are reflected in the proteome. We employed an experimental approach in which experimental and control cell lines were induced with doxycycline, followed by stable isotope labeling with amino acids in cell culture (SILAC) analysis. Experimental cells were grown in SILAC light medium, and control cells were grown in SILAC heavy medium containing C^13^-labeled lysine and arginine. Cell lines were grown for 16 days following doxycycline induction, followed by proteolytic digest and quantification of relative protein abundance by mass spectrometry approaches (Figure 4A). The experiment was performed in duplicate, enabling statistical analysis of the resulting differential protein abundance data. The replicates detected 2138 proteins in common, providing SILAC heavy/light ratios for 1081 (Figure 4B, 4C). Assessment of the reproducibility of SILAC heavy/light ratios measured between protein quantitation replicates revealed a strong correlation (0.92, *p*-value < 2.2E-16), validating the robustness and reproducibility of this method (Figure 4D).

We assessed whether changes in protein abundance preferentially impact specific gene ontologies. We extracted gene identifiers for the top 0.1 quantile of differentially-expressed proteins, and used this list to perform functional annotation enrichment analysis and term clustering using the DAVID functional annotation database [31] (Figure 4E, Supplementary Table 5). This analysis reveals several preferentially impacted term clusters. Notably, this analysis revealed a strong depletion of the ribosomal translational machinery (cluster #1) and a general up-regulation of mitochondrial proteins (cluster #2).

Sub-analysis of the proteins mapping to the translation-related cluster #1 revealed strong down-regulation of both cytosolic and mitochondrial ribosome structural components, as well as translation factors, but an increase in solute transporters responsible for shuttling glutamate, aspartate, citrate, and TCA cycle dicarboxylic acids across the mitochondrial membrane (Figure 4F). Previously, it has been reported that degradation of mature ribosomes is a hallmark of cellular autophagy, suggesting that autophagy may be activated in the context of SDHC loss [34].

Sub-analysis of proteins mapping to mitochondria-related cluster #2 revealed disparate effects upon several different classes of mitochondrial proteins (Figure 4G). Down-regulated groups include constituents of the mitochondrial ribosome and electron transport chain complex I and complex III. Protein groups that are strongly identified as up-regulated include TCA cycle, electron transport, fermentation, fatty acid metabolism, antioxidant defense, and solute transport across mitochondrial membrane. Intriguingly, we have previously shown that inhibition of alcohol dehydrogenase is synthetically lethal with SDH loss in budding yeast, and that the same is true for HEK293 cells knocked-down for SDHB [28]. This observation, together with the observed up-regulation of fermentation components LDHB and ALDH2 in the SDHC-loss iMEF cell culture model, suggests that SDH-deficient cells may become addicted to fermentation, which may represent a metabolic vulnerability amenable to drug targeting. We pursued this possibility.

### Synthetic lethal testing of LDHA and PCX depletion in the context of SDHC loss

**Figure 5:**
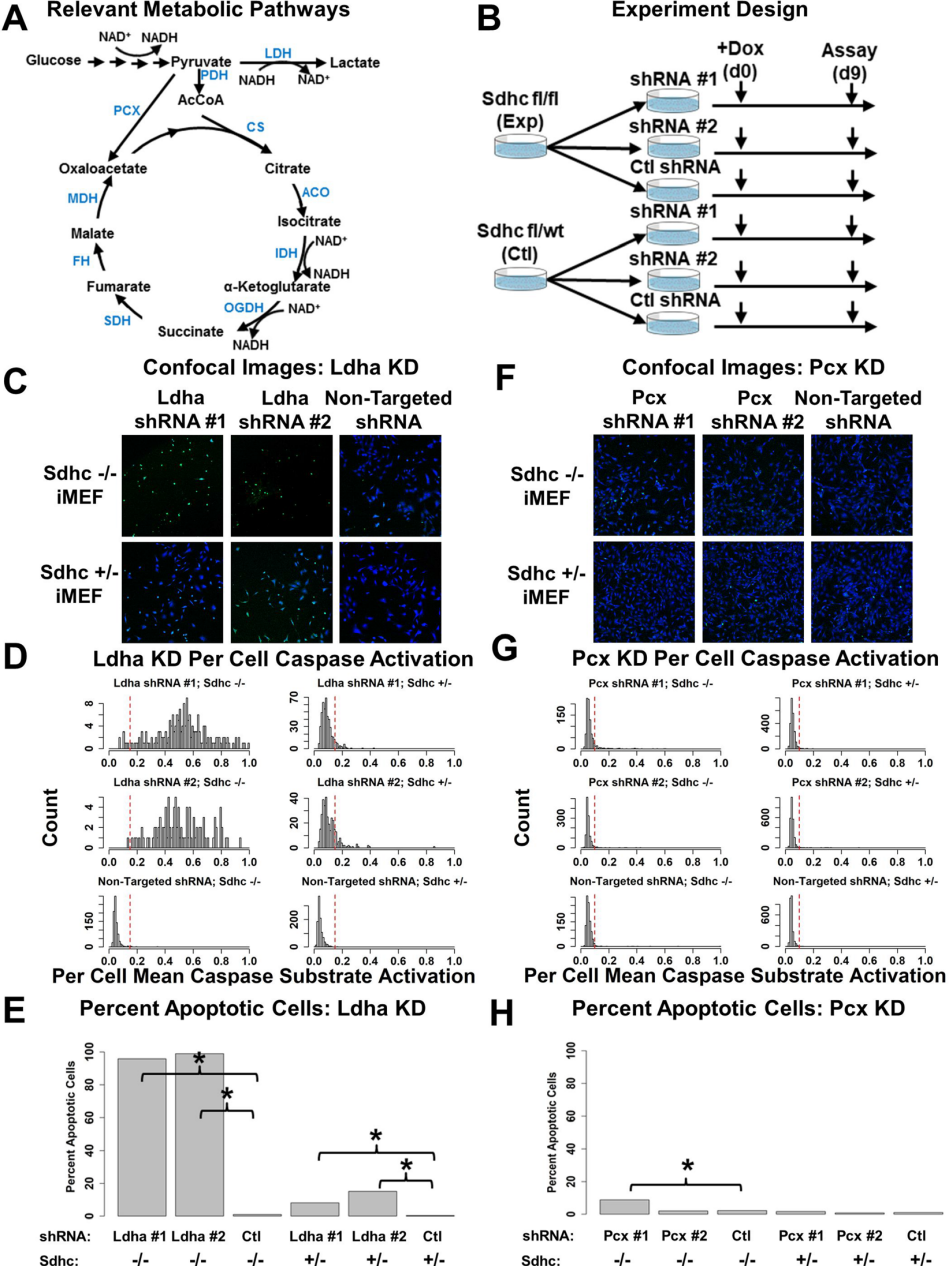
Testing synthetic lethality of LDHA and PCX depletion in the context of SDHC loss (**A**) Schematics of relevant metabolic pathways, including glycolysis and the TCA cycle. (**B**) Diagram of experimental design for synthetic lethal testing. Experimental (*Sdhc* fl/fl) and control (*Sdhc* fl/wt) iMEFs are transduced with shRNA-expressing lentiviruses to generate stable knockdowns of LDHA or PCX. Stable knockdown lines are then treated with doxycycline to induce *Sdhc* gene rearrangement and then assessed for apoptosis induction at day 9 post doxycycline initiation. (**C**) Representative confocal microscopy images from LDHA knock-down experiment. (**D**) Quantitation of per cell mean caspase substrate activation, derived from CellProfiler automated image analysis of LDHA knock-down experiment. Vertical red line indicates threshold caspase intensity for classifying a cell as apoptotic. (**E**) Quantitation of percent of total cells found to be apoptotic in LDHA knock-down experiment. Brackets and asterisks indicate comparisons found to be statistically significant by Chi-Sq test (*p*-value < 0.05). (**F**) Representative confocal microscopy images from PCX knock-down experiment. (**G**) Quantitation of per cell mean caspase substrate activation, derived from CellProfiler automated image analysis of PCX knock-down experiment. Vertical red line indicates threshold caspase intensity for classifying a cell as apoptotic. (**H**) Quantitation of percent of total cells found to be apoptotic in PCX knock-down experiment. Brackets and asterisks indicate comparisons found to be statistically significant by Chi-Sq test (*p*-value < 0.05).

SDH-deficient cells are thought to increasingly rely on the activity of LDHA for regeneration of NAD+ and PCX for synthesis of aspartate (Figure 5A). We therefore investigated the effects of LDHA and PCX inhibition using *Sdhc* fl/fl (experimental) and *Sdhc* fl/wt (control) iMEFs as models of SDH-deficient PPGL and normal cells, respectively.

We first tested whether SDH-deficient cells are more sensitive to LDHA knockdown than are cells with intact SDH activity. iMEFs were transduced with two independent short hairpin RNA (shRNA)-expressing lentiviruses to generate stable LDHA knockdown iMEF lines (Figure 5B). Controls were generated by transducing corresponding *Sdhc* fl/fl and control *Sdhc* fl/wt cells with a non-targeted shRNA-expressing lentivirus. LDHA depletion was confirmed by quantitative Western blot analysis showing dramatic reduction in protein levels (Supplementary Figure 4A, 4B). The cells were then treated with doxycycline to trigger *Sdhc* gene rearrangement and subsequent loss of SDHC protein. Consistent with synthetic lethality upon metabolic rewiring of SDH loss cells, LDHA deficiency resulted in marked cleavage of caspase substrate as a marker for apoptosis in SDHC-deficient cells on day 9 post doxycycline initiation (Figure 5C, 5D). In contrast, apoptosis was rarely observed in LDHA depleted control iMEFs and no apoptosis was observed in non-targeted Sdhc-deficient and control iMEF cell lines (Figure 5E). These results demonstrate that loss of LDHA activity is synthetically lethal with SDH loss in iMEFs, and confirm the essential role of LDHA in SDH-null cell survival, and echoes previous results obtained in SDH-loss yeast cells and a SDHB-knockdown mammalian cell model [28].

To explore the effects of PCX knockdown in SDH-loss cells, both *Sdhc* fl/fl and *Sdhc* fl/wt iMEFs were transduced with two independent shRNA-expressing lentiviruses and one non-targeted shRNA-expressing lentivirus to generate three stable PCX knockdown cell lines (Figure 5B). PCX depletion was confirmed via quantitative Western blot analysis of the PCX knockdown cell lines (Supplementary Figure 4A, 4B). Cells were then treated with doxycycline to induce *Sdhc* gene rearrangement and evaluated for caspase substrate cleavage as a marker of apoptosis on day 9 post doxycycline initiation (Figure 5F, 5G). Interestingly, apoptosis levels were not statistically different between experimental Sdhc fl/fl and control Sdhc fl/wt iMEFs indicating that SDH loss is not synthetically lethal with PCX knockdown in this iMEF model (Figure 5H). This result suggests that the prior observation of PCX synthetic lethality with SDH loss in a different model is not a universal characteristic of SDH-loss cell lines [30].

### Testing chemical inhibitors of LDH in *Sdhc*-null iMEF model

**Figure 6:**
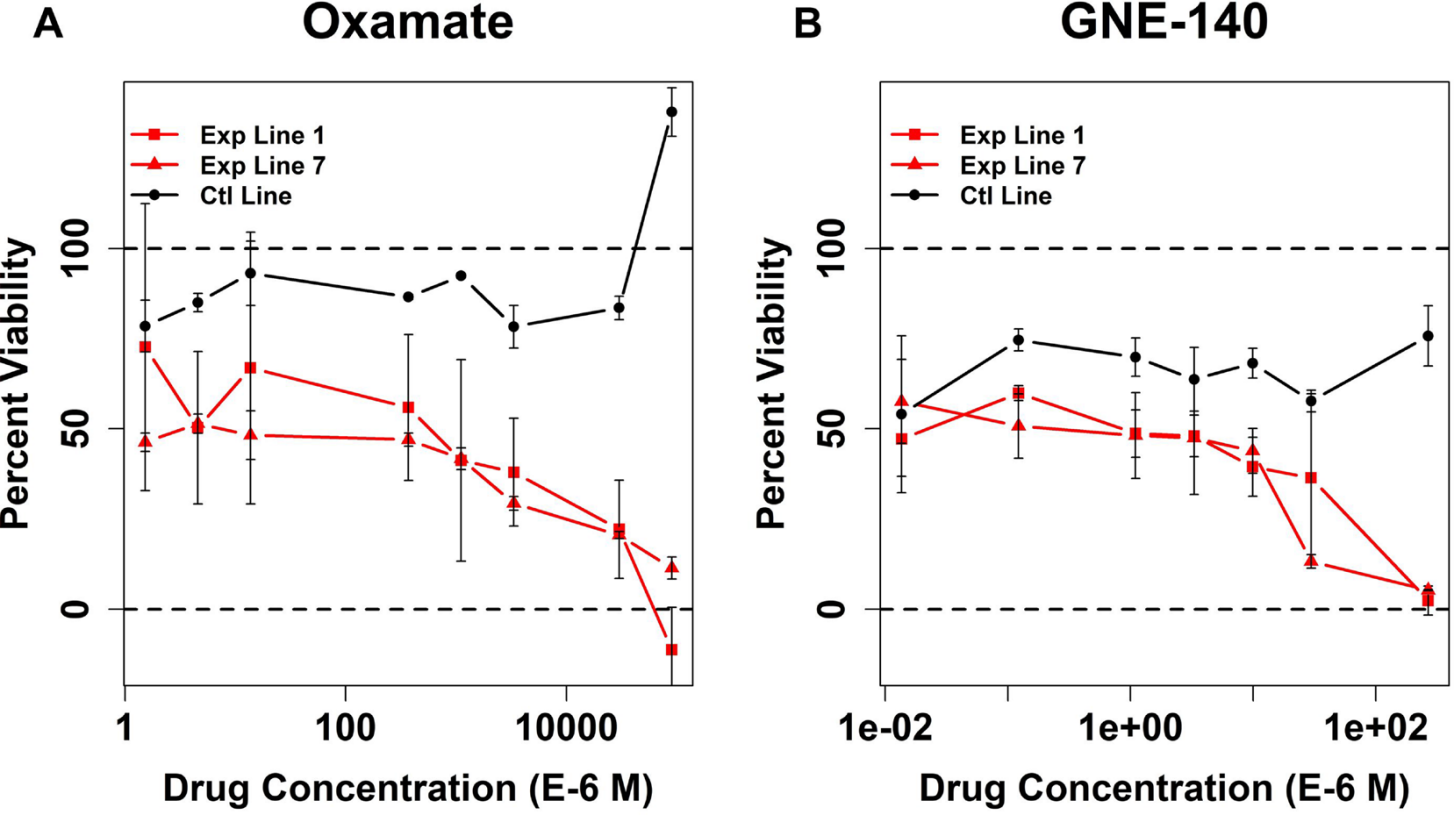
Testing LDH chemical inhibitors for lethality in Sdhc-loss context Y-axis of plots reflects relative percent reduction of Alamar Blue for drug-treated cells relative to vehicle-treated cells of the same line. X-axis shows drug concentration on a log_10_ scale. Error bars show standard deviations calculated over *N* = 3 experimental replicates. Welch two sample *t*-test comparing viability of experimental and control lines at highest oxamate concentration yields *p*-values of 0.01 and 0.01 for experimental lines 1 and 7, respectively. Welch two sample *t*-test comparing viability of experimental and control lines at highest GNE-140 concentration yields *p*-values of 0.03 and 0.07 for experimental lines 1 and 7, respectively.

The observation of synthetic lethality of LDHA loss and SDH loss suggested that inhibition of LDH enzymatic activity by commercially-available small molecules could result in preferential toxicity to SDHC-loss cells. To assess this, we performed drug titration assays using stable SDHC loss and control lines measuring differential cell viability between drug-treated and vehicle-treated cells with a standard assay. We tested two commercially-available small molecule LDH inhibitors: oxamate and GNE-140 [35]. Both compounds showed robust cell killing in experimental cell lines (Figure 6). Importantly, both compounds were relatively non-toxic to control cells, even at high doses. This suggests that LDH inhibitors may have a large therapeutic window. These results confirm the conclusions of the lentiviral shRNA-mediated knockdown experiments, suggesting metabolic reliance upon LDH activity and vulnerability to LDH inhibition in SDHC loss cells.

## DISCUSSION

### Development and phenotypic characterization of SDHC-loss iMEF model

In the current work, we developed a tet-inducible iMEF model with silencing gene rearrangement of the *Sdhc* floxed allele driven by doxycycline-dependent expression of cre-recombinase from a tet-inducible promoter. Using this model and an isogenic *Sdhc* fl/wt control cell line, we verified that our cell lines displayed robust *Sdhc* gene rearrangement, SDHC and SDHB protein loss, and accumulation of succinate following induction with doxycycline.

### Differential gene expression analysis

We show here that SDHC loss in iMEF cells results in differential expression of 161 genes, and additionally show that altered gene expression is negatively correlated with original gene expression value in the control cell line. We additionally show that genes having a baseline expression value < 6 RPKM tend to increase in expression in the context of SDHC-loss, while genes having expression > 6 RPKM tend to decrease in expression. This finding that patterns of SDHC-loss transcriptional change correlate with baseline expression values in normal cells has not previously been reported, and points to distinct transcriptional activating and repressing effects that operate on these gene subsets in the context of SDHC-loss. The basis for these disparate effects is not clear.

We also show a preferential impact of SDHC-loss on several key gene ontologies involved in antiviral response, innate immunity, disulfide bond formation, and extracellular matrix production. It is unclear to what extent altered expression of these cellular components accounts for a malignant phenotype in human PPGL, but it is intriguing to consider that altered expression of these components, and especially extracellular matrix components, could facilitate altered adhesion of a cell to its host tissue and enable increased motility, properties that are likely essential for malignant invasion of local structures and metastasis.

### Genome-wide DNA methylation analysis

Here we show that CpG site methylation information is sufficient to correctly classify specimens, and that this analysis intriguing suggests that the day 0 experimental specimen is more similar to the control specimens than to experimental specimens at days 5–20. This suggests that significant differences in methylation state are driven by SDHC loss, and that these changes are relatively stable across the time-course.

A major finding in our analysis is that the initial methylation value for individual CpG sites and promoters is correlated with the methylation difference observed upon SDHC loss. Sites with initially low methylation values tend to become hypermethylated, consistent with the currently-accepted model of succinate-induced inhibition of Tet enzyme activity. Counterintuitively, for sites with initially high levels of methylation, the opposite pattern is true. Sites with initially high levels of cytosine methylation tend to become hypomethylated in response to SDHC loss. We further show that this pattern is true both at the level of individual CpG sites and at the promoter level. The basis for this global hypomethylation of sites that are initially hypermethylated is unclear. SDHC loss in this cell model is therefore best described as inducing derangement of DNA methylation. This result differs importantly from the prior characterization of a hyper-methylator phenotype upon SDH loss. Our data would have supported such a hypermethylator phenotype if we had focused only on DNA sites that had initial low methylation.

We also demonstrate that SDHC-loss-attributable patterns of promoter methylation difference correlate both with patterns of SDHx-loss in human PPGL tumors, and also with patterns of SDHB-loss in imCCs, previously described by others. Perhaps unsurprisingly, we find that the difference correlations between SDHB-loss imCCs and SDHx-loss human PPGL tumors is higher than observed in the iMEF comparison, indicating that SDHB-loss imCCs may be a better model for approximating the epigenetic state and changes of human tumors. However, we were nonetheless successful in identifying a conserved subset of gene promoters that are hypermethylated across species and tissue types in the context of SDH-loss. This indicates that some aspects of global epigenomic derangement are conserved in response to SDH loss.

### Global proteomic characterization of SDHC-loss iMEFs

Here we also present a global characterization of proteomic changes that occur in response to SDHC loss. This analysis features an isotopic labeling strategy and mass spectrometry quantitation of relative protein abundance between experimental and control cell lines, and was found to be highly reproducible between replicate experiments. This analysis revealed the unprecedented finding that translation-related proteins are dramatically down-regulated in the context of SDHC loss, suggesting an autophagy stress response. We also show that mitochondrial proteins are dramatically impacted, and that specific differences correlate with the pathways in which the various proteins are involved. Importantly, we show up-regulation of fermentation pathway components, including LDHB and ALDH2. Taken in conjunction with our previous published work showing that inhibition of alcohol dehydrogenase in yeast is synthetic lethal with SDH loss, this is additional evidence suggesting that fermentation is up-regulated in SDH-loss context. This suggested a potential metabolic vulnerability amenable to drug targeting.

### Metabolic vulnerabilities in SDH loss

SDH-loss PPGL exhibits an increased dependence on glycolysis for ATP generation and production of carbon skeletons for growth. In the absence of SDH, there is evidence for linear metabolic pathways rather than a conventional TCA cycle [17, 33, 36]. Thus, metabolic reprogramming in SDH-deficient tumors represents a potential therapeutic target. A prior high-throughput drug screen in SDH-loss yeast pointed to alcohol dehydrogenase, the fungal equivalent of mammalian lactate dehydrogenase, as synthetically lethal with SDH loss [28]. Here we used shRNA depletion of LDHA and PCX to probe synthetic lethality in the iMEF cell model of hereditary SDH-loss PPGL. In *Sdhc* fl/fl cells, LDHA inhibition showed profound synthetic lethality with SDH loss, with induction of robust cell death via apoptosis. Induced apoptosis in *Sdhc* fl/wt cells was significantly lower. In contrast, PCX knockdown did not exhibit synthetic lethality with SDH loss in these cells. Compared to previously published data [30] these data suggest differences in the underlying metabolism of the different cells lines studied. We conclude that *Sdhc* fl/fl iMEF cell lines show increased reliance on glycolysis for cell survival and proliferation. We further confirm that LDH is a point of metabolic vulnerability in SDH loss by performing drug studies examining the viability of experimental and control cell lines in the presence of varying amounts of oxamate or GNE-140, two commercially available LDH inhibitors. These studies revealed that chemical inhibition of LDH in SDH loss cells is lethal, whereas similar drug concentrations in the control cell line had little effect. Although it is doubtful that currently available LDH inhibitors such as oxamate or GNE-140 would be suitable for application in animals or humans due to limited potency, our studies suggest a rationale for further development of more potent molecules. In summary, our data on genetic synthetic lethality and drug testing suggest that SDH-deficient PPGL may be especially sensitive to lactate dehydrogenase inhibitors.

## MATERIALS AND METHODS

### Generation of SDHC-loss iMEF lines

A tet-inducible conditional knockout SDHC mouse was bred using a gene trap construct obtained from the Sanger Institute and converted to a conditional floxed Sdhc exon 4 construct by FLP recombination. In this model, silencing gene rearrangement of the *Sdhc* floxed allele is driven by doxycycline-dependent expression of Cre recombinase from a tet-inducible promoter.

Through routine mouse breeding, we generated *Sdhc* fl/fl;R26M2rtTA/M2rtTA animals (where fl refers to a construct with *Sdhc* exon 4 flanked by Cre recombination sites and R26M2rtTA refers to the reverse tetracycline trans-activator) and *Sdhc* flwt;TetOcre animals (where wt indicates the *Sdhc* wild type allele and TetOcre indicates tet-inducible cre-recombinase). We then crossed Sdhcfl/fl;R26M2rtTA/M2rtT animals with Sdhcfl/wt;TetOcre animals to yield R26M2rtTA/wt;TetOcre;Sdhcfl/fl (experimental) and R26M2rtTA/wt;TetOcre;Sdhc fl/wt (control) genotypes and established MEF cell lines from day 13 mouse embryos. Genotypes of cell lines were characterized via PCR and cell lines were immortalized via SV40 virus.

### Induction of *Sdhc* exon 4 rearrangement and time-course serial sampling

The established *Sdhc* fl/fl cell culture model and an isogenic *Sdhc* fl/wt control line were treated with doxycycline (1 µg/mL) to induce robust *Sdhc* gene rearrangement. Cell pellets used for experiments were derived at time of passaging by trypsinization, quenching with FBS, centrifuging to gently pellet the cells (500 rpm for 5 min at 4°C), and washing 2X with PBS prior to freezing at −80°C.

### *Sdhc* rearrangement PCR

Genomic DNA was extracted from isolated wet cell pellets using the Qiagen Blood and Tissue kit (Qiagen cat# 69504). 100–200 ng of DNA was amplified using PCR primers LJM-4429 (CTTAGAACTGATCCCCTGCCC) and LJM-5125 (CCTGGAACTAGAATTATTGATGGATG) at 300 nM concentration and PCR Master Mix (Thermo Fisher cat# K0171). Thermal cycling parameters include 15 minutes at 95°C, 38X(30 seconds at 95°C, 90 seconds at 58°C, 2 minutes at 72°C), 10 minutes at 68°C, followed by indefinite hold at 4°C. *Sdhc* floxed allele yields a product of 1774 bp, and the recombined allele gives an expected product of 560 bp.

### SDHC and SDHB Western blot analysis

Loss of SDHB and SDHC proteins was confirmed by Western blot. Briefly, cells were lysed in RIPA buffer (50 mM Tris-HCl, 5 mM EDTA, 150 mM NaCl, 0.1% SDS, 0.5% DOC, 1% NP-40) on ice with gentle pipetting and vortexing. Lysates were centrifuged at 15,000 × g for 5 min to pellet debris, and protein concentration of the supernatant was quantified by BCA assay. 20 micrograms of total protein was then combined with reducing agent and LDS buffer and heated to 90°C for 5 minutes to denature the proteins. Samples were loaded onto a 10% bis-tris gel (NuPAGE, cat# NP0301BOX) and run at 130 V for 45 minutes using 1X MES-SDS as the running buffer. Blot transfer was conducted using PVDF membranes and NuPAGE transfer buffer containing 20% methanol at 30V for 90 minutes at 4°C. Transferred proteins were visualized on the membrane using Ponceau S stain, and membranes were cut using a razor blade. Membranes were then blocked for 1 hr at room temperature using blocking buffer consisting of 100 mL TBST and 3 g nonfat dry milk, and then washed 3X 5 min with 1X TBST. Solutions of primary antibodies were then prepared in antibody dilution buffer (2.5 mL 4% BSA, 250 microliters 0.5% NaAzide, 7.5 mL TBST). Antibodies used in this analysis include a SDHC polyclonal IgG antibody raised in rabbit (Santa Cruz Biotechnology, cat# sc-67256 (M-169), 1:500 dilution), a SDHB polyclonal IgG antibody raised in rabbit (Invitrogen cat# PA5-21587, 1:1000 dilution), and actin polyclonal IgG antibody raised in rabbit (Sigma, cat# A2066, 1:500 dilution). The next day, primary antibody solutions were removed and membranes were washed 3X 5 minutes with TBST at room temperature. Membranes were then incubated with secondary antibody (1:10,000 dilution of anti-rabbit HRP antibody into blocking buffer) for 1 hr at room temperature. Membranes were then washed 3X 5 min with TBST, and then incubated with ECL Western blot substrate for 5 min at room temperature prior to imaging substrate fluorescence on the Typhoon fluorimeter. Band intensities of digital images were quantified using ImageQuant software, normalizing LDHA and PCX levels to ACTB.

### TCA cycle metabolomics

Isolated cell pellets from the described time-course experimental design underwent targeted analysis of TCA cycle metabolites by GC-MS in the Mayo Clinic Metabolomics Core Facility. Quantified metabolite values were normalized to total protein abundance prior to downstream analysis and data visualization. The described analysis was performed in triplicate for each time point and cell line.

### Doubling time quantitation

Cell population doubling time for experimental and control iMEF lines was assessed as a function of time following induction of *Sdhc* gene rearrangement with doxycycline. The method of quantifying doubling time involved capturing several sets of images of marked positions on a T75 cell culture flask using a phase contrast microscope, with ∼24 h time difference between image sets. For each set of images, the number of cells in the respective images was determined by automated detection of cell outlines in CellProfiler [34]. Cell population doubling time was calculated using the quantified cell numbers at the two time points and known time interval between measurements using the equation,

$$T_{d}=\left(t_{2}-t_{1}\right)\left(\frac{\log\;2}{\log\frac{n_{2}}{n_{1}}}\right)$$

where n_1_ and n_2_ are the cell numbers quantified at times t_1_ and t_2_, respectively. For each time point and cell line, this calculation was repeated for at least four pre-marked imaging fields. Results are presented as mean and standard deviation of the obtained measurements.

### RNA-seq gene expression analysis

Cell pellets for *Sdhc* fl/fl and *Sdhc* fl/+ iMEF lines collected over the time series experiment were subjected to RNA extraction using the Qiagen RNeasy kit. Purified RNA was the submitted to the Mayo Clinic Medical Genome Facility for indexing and preparation of deep sequencing libraries using the Illumina TruSeq mRNA v2 kit, followed by deep sequencing on a HiSeq 4000 instrument, multiplexing 8 samples per lane and performing 100 sequencing cycles. Following sequencing, reads belonging to individual experiments were deconvoluted on the basis of unique sequence barcodes. Deconvoluted sequence read data for individual experiments are available from the NCBI sequence read archive (SRA) under identifier SRP117182.

Paired end sequence reads were then aligned to the mm9 mouse reference genome using the Bowtie fast read aligner [28] and the Mayo Clinic Research Computing Facility Beowulf-style Linux cluster. SAM files were then converted to BAM file format using SAMtools [37], and FPKM quantitation of individual transcript abundance was performed using R package systemPipeR [38], available through the Bioconductor project. The resulting processed gene expression datasets are available via NCBI Gene Expression Omnibus (GEO) under entry GSE103662.

### RRBS analysis of genome-wide DNA methylation patterns

Cell pellets for *Sdhc* fl/fl and *Sdhc* fl/wt iMEF lines collected over the time series experiment were subjected to DNA extraction using the Qiagen Blood and Tissue kit. Purified genomic DNA was then submitted to the Mayo Clinic Medical Genome Facility for subsequent bisulfite conversion, indexing and preparation of paired-end deep sequencing libraries, followed by deep sequencing on a HiSeq 4000 instrument, multiplexing 8 samples per lane and performing 100 sequencing cycles. Following sequencing, reads belonging to individual experiments were deconvoluted on the basis of unique sequence barcodes. Deconvoluted sequence read data for individual experiments are available from the NCBI sequence read archive (SRA) under identifier SRP117088.

Sequence reads were then aligned to the mm10 mouse reference genome using the Bismark bisulfite read mapper [39] and the Mayo Clinic Research Computing Facility Beowulf-style Linux cluster. Methylation calling was performed using the Bismark methylation extractor utility, and CpG context-specific methylation status of individual sites was extracted using the Bismark bismark2bedGraph utility.

Bismark .COV files were then used in downstream filtering and analysis performed in R using the package RnBeads [40]. In brief, the data was filtered to remove sex chromosomes, CpG sites with exceptionally high coverage, CpG sites with no methylation information, and CpG sites having exceptionally low standard deviation in beta value across samples. Differential methylation analysis at the CpG site level was performed in RnBeads, grouping all *Sdhc* fl/wt samples into the control group, and including *Sdhc* fl/fl samples, except for the day 0 sample, in the experimental group. Beta values for individual CpG sites, as well as averaged beta values for CpG sites mapping to annotated GpG islands and promoters (1.5 kb upstream and 500 bp downstream of annotated TSS) were exported for downstream exploratory analysis. The resulting processed DNA methylation datasets are available via NCBI Gene Expression Omnibus (GEO) under entry GSE103609.

Average differences in site-specific, CpG island-specific, and promoter-specific methylation beta values were calculated by subtracting the methylation values of the *Sdhc* fl/wt iMEF line (control) from the *Sdhc* fl/fl iMEF line (experimental).

### Correlative analysis of iMEF DNA methylation patterns with imCCs and human PPGL tumors

Processed Bismark .COV files describing DNA methylation change in SDHB-loss immortalized mouse chromaffin cells (imCCs) were downloaded from NCBI GEO entry GSE43298. This dataset has been described previously [33]. Data were handled in RnBeads using the same methods as used to process iMEF RRBS data to produce methylation beta value quantitations. Average differences in promoter-specific methylation beta values were calculated by subtracting the mean methylation values of the SDHB-wt imCC line (control) from the SDHB-loss imCC line (experimental).

Datasets measuring DNA methylation in biopsied human PPGL tumors via Illumina HumanMethylation450 BeadChip were obtained from ArrayExpress under entry E-GEOD-43298. This dataset has also been described previously [33]. Average beta values for SDHx tumors and all other tumors were calculated in R. Average differences in promoter-specific methylation beta values were calculated by subtracting the mean methylation values the CpG island nearest to a given TSS of the non-SDHx PPGL tumors (control) from the SDHx PPGL tumors (experimental).

Correlative analysis of methylation differences observed in SDHC-loss iMEFs, SDHB-loss imCCs, and SDHx-attributable PPGL tumors was performed in R. Analysis of functional term enrichment in subset of genes observed to have promoter hypermethylation (beta difference > 0.05) across all three contexts was performed using the DAVID functional annotation database [31].

### Generation and characterization of stable LDHA and PCX knockdown lines

The lentiviral non-targeted TRC1and1.5 shRNA and shRNA plasmids against LDHA and PCX were purchased from Sigma-Aldrich. Clone names for LDHA knock-down constructs were NM_010699.1-1484s1c1 (LDHA8), NM_010699,1-434s1c1 (LDHA9), NM_010699,1-537s1c1 (LDHA10), NM_010699.1-603s1c1 (LDHA11), and NM_010699.1-822s1c1 (LDHA12). Clone names for PCX knock-down constructs were NM_008797.1-1057s1c1 (PCX13), NM_008797.1-1456s1c1 (PCX14), NM_008797.1-190s1c1 (PCX15), NM_008797.1-2986s1c1 (PCX16), and NM_008797.1-3805s1c1 (PCX17). To generate stable knockdown LDHA and PCX cell lines, lentiviral infections were carried out as previously described [20]. Transduced cells were incubated with medium containing 35 µg/mL puromycin (selection medium) for 48 h before being used for further experiments. Loss of LDHA and PCX were confirmed via Western blot analysis.

### Cell lysis and protein digestion

SDH WT and KO cells were lysed with buffer containing 9M urea in PBS (pH 7.2), 50 mM nicotinamide, 25 mM sodium butyrate and HALT protease inhibitor cocktail (Thermo Fisher). Proteins were reduced with 5 mM TCEP and alkylated with 5 mM iodoacetamide as previously described [41]. Equal amounts of heavy- and light-labeled proteins were mixed and then diluted by six-fold with PBS. Proteins were digested with trypsin (enzyme to substrate ratio 1:50, w/w) overnight at room temperature. Proteins were digested again with trypsin (enzyme to substrate ratio 1:100, w/w) for three h at room temperature to complete digestion. Peptide desalting was performed with the Sep-pack C18 cartridge (Waters) according to manufacturer’s directions.

### SCX fractionation

Peptides were pre-fractionated and desalted according to a previously described method [42]. For pre-fractionation, peptides were loaded into stage-tips packed with Empore Cation Exchange-SR membranes (3 M). The membrane was washed with 0.1% formic acid and the peptides were successively eluted with six buffers containing 20% acetonitrile (v/v), 0.1% formic acid (v/v) with concentrations of 50, 75, 125, 200, 300 and 500 mM NH_4_OAc. The peptides from each fraction were collected and desalted with C18 Stage-tips and subsequently dried in a Speed-Vac (Thermo Fisher).

### LC-MS/MS measurement

Desalted peptides were resolubilized with HPLC buffer A (0.1% formic acid, v/v) and loaded onto a self-prepared C18 column (15 cm × 75 µm, ReproSil-Pur Basic C18, 2.5 µm, Dr. Maisch GmbH). Peptides were analyzed with a Proxeon Easy nLC 1000 Nano-UPLC system and an Orbitrap Fusion mass spectrometer (Thermo Fisher). Peptides were separated with a 56-min linear gradient of 5–30% acetonitrile (v/v) in 0.1% formic acid (v/v) with a 200 nL/min flow rate. Full MS was acquired at a resolution of 60,000 and covered a mass range of m/z 300–1500. The MS data were acquired in a data-dependent manner, giving priority to the most intense precursor ions. Precursor ions were fragmented with collision dissociation (CID) with 35% collision energy for ion trap detection.

### Raw proteomic data processing

Raw MS/MS spectra data was processed by MaxQuant (v 1.4.1.2) for protein quantification against the Uniprot mouse database (downloaded on 2013/09/27 with a total of 43310 sequences). The fixed cysteine carbamidomethylation modification and the variable modifications of methionine oxidation and protein N-terminal acetylation were specified. The proteolytic enzyme, trypsin, was selected, permitting a two missing cleavages. Multiplicity was set to two. R6 and K6 were specified as heavy amino acid labels with a maximum of three labeled amino acids per peptide. Default values were included for the rest of the search parameters. The precursor ion and fragment ion mass tolerance was set at 4.5 ppm and 0.5 Da respectively. The database search was filtered to achieve a 1% False Discovery Rate (FDR) at peptide, protein and modified site level. The minimum Andromeda score for modified peptides was set at 40. The mass spectrometry proteomics data have been deposited to the ProteomeXchange Consortium via the PRIDE [43] partner repository with the dataset identifier PXD007874.

### Testing synthetic lethal interaction of SDHC with LDHA and PCX

Confocal microscopy images from LDHA and PCX knockdown experiments were assessed in replicates on day 9 post doxycycline initiation. CellEvent caspase 3/7 activity detection reagent (catalog number C10423, from Invitrogen; final 5 µM caspase reagent) was added to the media in the well of a 96-well plate. The plate was incubated for 30 min at 37°C. Media were aspirated and 3.7% formaldehyde solution was added to the wells. The plate was incubated at room temperature for 15 min. Formaldehyde solution was aspirated and DAPI solution (catalog number 10236276001, 10 mg; final 5 µg/mL) was added to the wells. The images were obtained with a confocal microscope. Quantitation of per cell mean caspase substrate activation was derived from CellProfiler automated image analysis. Quantitation of percent of total apoptotic cells in LDHA and PCX knock-down experiments was compared by Chi-Sq test to detect statistical significance.

### Generation of stable SDHC-loss iMEF lines

Following induction of *Sdhc* gene rearrangement in *Sdhc* fl/fl and *Sdhc* fl/wt iMEF lines for 5 d, cells were diluted into DMEM containing penicillin/streptomycin antibiotics (0.5 mg/mL), non-essential amino acids (100 micromolar each of glycine, alanine, asparagine, aspartic acid, glutamic acid, proline, and serine), sodium pyruvate (1 mM), and HEPES buffer (10 mM), and then seeded into 96 well plates at a low density so as to achieve single cells per well. Plates were then incubated at 37°C for 2 weeks prior to visually screening wells to identify clonal populations of cells. Clones were then progressively expanded into 12-well plates and T25 flasks, using the same media. Small numbers of cells from each clone were subjected to DNA extraction and PCR analysis of *Sdhc* gene rearrangement status. Clones demonstrating homogenous *Sdhc* rearrangement were subsequently expanded and used in chemical inhibitor assays.

### Testing effects of LDHA chemical inhibitors in SDHC-loss iMEF lines

For drug studies testing metabolic vulnerability of LDH inhibition, 13.5E6 cells from stable SDHC loss and control iMEF lines were plated into wells of a 96 well plate in 100 microliters of phenol red-free DMEM media containing penicillin/streptomycin antibiotics (0.5 mg/mL), non-essential amino acids (100 µM each of glycine, alanine, asparagine, aspartic acid, glutamic acid, proline, and serine), sodium pyruvate (1 mM), and HEPES buffer (10 mM). Cells were allowed to adhere to the plate overnight prior to drug exposure. Oxamate was obtained from Sigma Aldrich. GNE-140 was kindly provided by Genentech, and has previously been described [35]. Oxamate was dissolved in water and GNE-140 was dissolved in DMSO. Drugs were then serially diluted and added to plates. The final concentration of DMSO in media of GNE-140-treated cells was 1%. Plates were then incubated at 37°C for 3 d, at which point 10 µL Alamar Blue (Thermo Fisher) cell viability reagent was added to each well. Plates were incubated for 36 h prior to taking absorbance measurements at 570 and 600 nm. Amount of reduced Alamar Blue was calculated using the obtained absorbance measurements and the following equation,

$$AR_{570}=A_{570}-\left(A_{600}\;XR_{o}\right)$$

where AR_570_ is the amount of reduced Alamar Blue, A_570_ and A_600_ are the absorbance measurements at 570 and 600 nm, respectively, and R_o_ (0.69) is the empirically-determined ratio of A_570_ and A_600_ absorbances for Alamar Blue in media with no cells. Percent difference in Alamer Blue reduction was then determined between drug-treated cells and vehicle-treated cells for each tested cell line.

## SUPPLEMENTARY MATERIALS FIGURES AND TABLES













## Abbreviations

PGL: paraganglioma
PCC: pheochromocytoma
PPGL: pheochromocytoma and paraganglioma
SDH: succinate dehydrogenase
SDHC: succinate dehydrogenase subunit C
SDHB: succinate dehydrogenase subunit B
SDHx: any succinate dehydrogenase subunit
LDH: lactate dehydrogenase
LDHA: lactate dehydrogenase form A
PCX: pyruvate carboxylase
