# Supplementary material for: Characterization and metabolic synthetic lethal testing in a new model of SDH-loss familial pheochromocytoma and paraganglioma
Source: Oncotarget. 2017 Dec 22;9(5):6109–27. doi: 10.18632/oncotarget.23639 (PMC5814199; doi:10.18632/oncotarget.23639)
Supplement: Supplementary file 1 [file oncotarget-09-6109-s001.pdf]

Characterization and metabolic synthetic lethal testing in a new model of SDH-loss familial pheochromocytoma and paraganglioma

SUPPLEMENTARY MATERIALS

Supplementary Table 1: Unfiltered DE genes. See Supplementary_Table_1

Supplementary Table 2: Filtered DE genes. See Supplementary_Table_2

Supplementary Table 3: RNA-seq DAVID functional enrichment analysis. See Supplementary_Table_3

Supplementary Table 4: RRBS DAVID functional enrichment analysis. See Supplementary_Table_4

Supplementary Table 5: SILAC protein quantification DAVID functional enrichment analysis. See Supplementary_Table_5

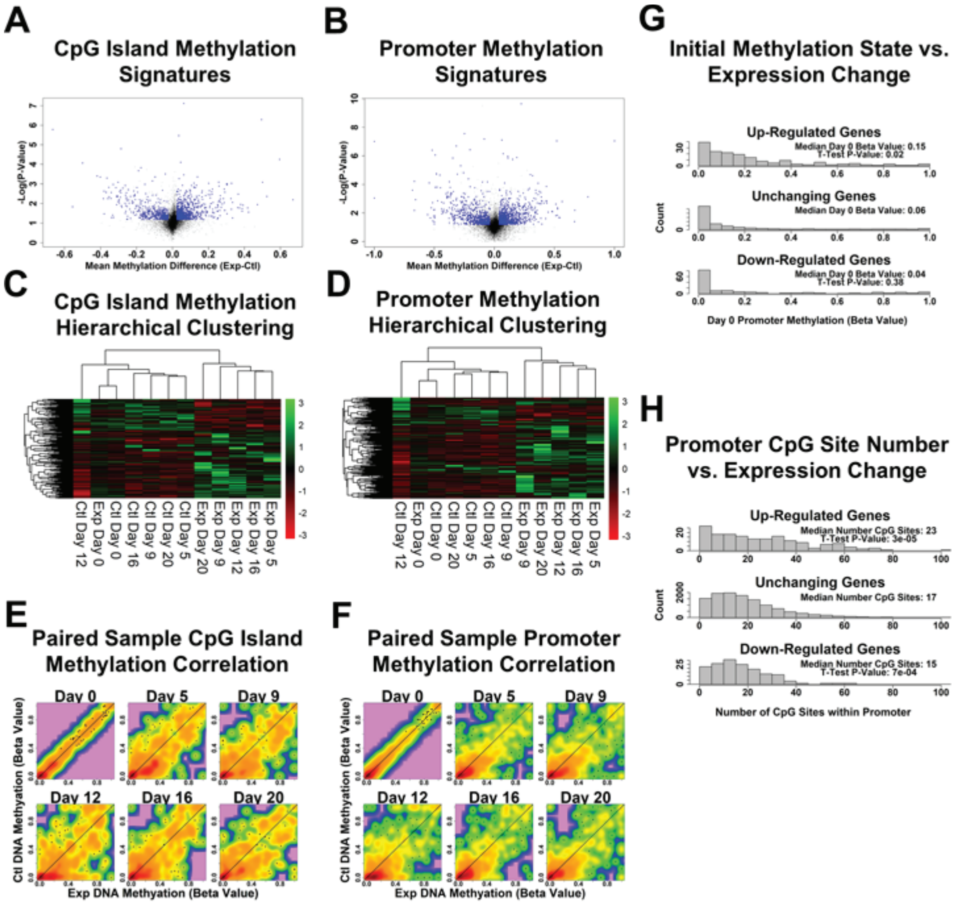

Supplementary Figure 1: Supplemental analysis of genome-wide methylation patterns in SDHC loss iMEFs. (**A**, **B**) Volcano plots showing CpG island and promoter mean methylation differences, respectively, versus –log(*p*-value) from RnBeads differential methylation analysis. Blue dots correspond to the top 0.1 quantile of the dataset, as quantified by combined rank. (**C**, **D**) Hierarchical clustering of samples based upon DNA aggregate methylation patterns at CpG islands and promoters, respectively. (**E**, **F**) Correlation heat maps showing the emergence of DNA methylation differences at CpG islands and promoters, respectively, between experimental and control iMEF lines following induction of SDHC loss with doxycycline. Only differences emerging after day 0 are shown. Colors correspond to data point density (red = high, green = intermediate, blue = low) (**G**) Correlative analysis of initial promoter methylation state (beta value at day 0) and gene expression change at 20 days post dox initiation. Up-regulated genes were identified as having $\log_2$(fold-change) > 1. Down-regulated genes were identified as having $\log_2$(fold-change) < –1. Indicated *T*-tests were calculated in reference to the unchanging subset of genes by comparing distributions of promoter day 0 methylation beta values. (**H**) Correlative analysis of promoter CpG site number and gene expression change at 20 days post dox initiation. Up-regulated genes were identified as having $\log_2$(fold-change) > 1. Down-regulated genes were identified as having $\log_2$(fold-change) < –1. Indicated *T*-tests were calculated in reference to the unchanging subset of genes by comparing distributions of promoter CpG site numbers.

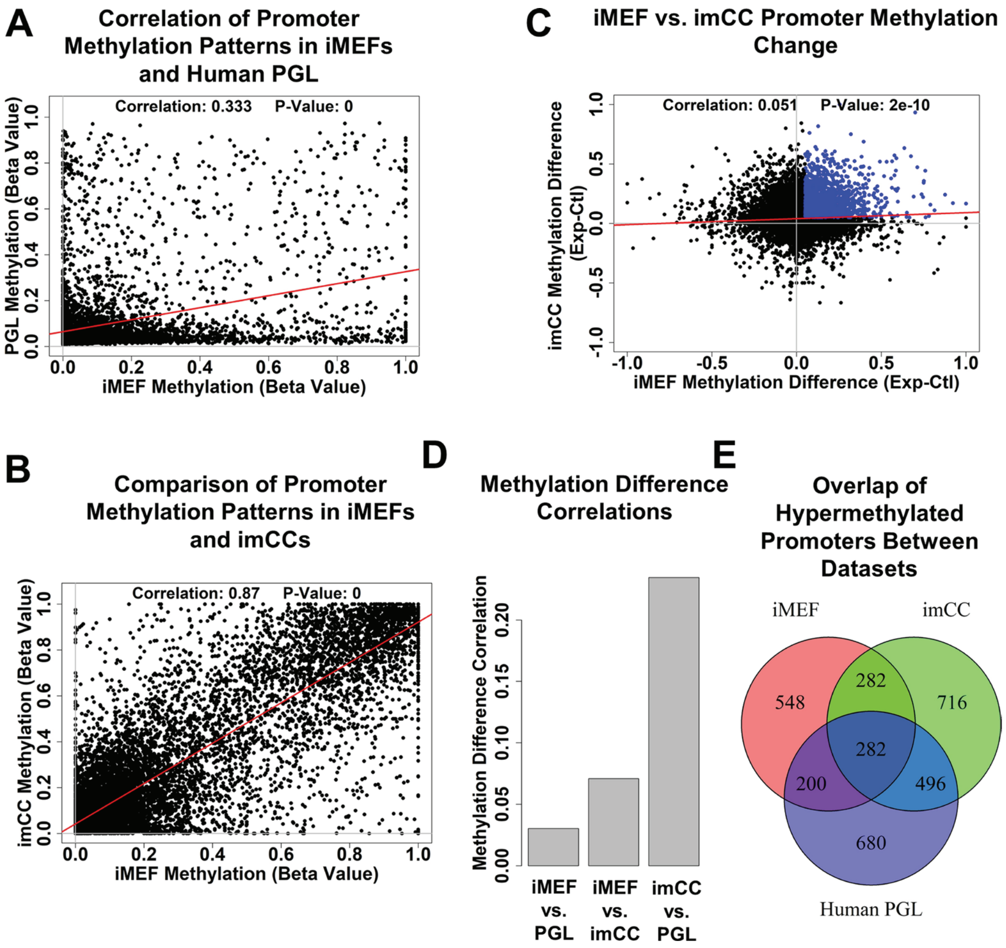

Supplementary Figure 2: Comparison of methylation patterns observed in SDHC-loss iMEFs, SDHB-loss imCCs, and SDHx PPGL tumors. (**A**) Correlation of promoter methylation patterns observed in normal iMEFs and PGL tumors. (**B**) Correlation of promoter methylation patterns observed in normal iMEFs and imCCs. (**C**) Comparison of SDHC-loss iMEF and SDHB-loss imCC promoter methylation changes genome-wide. Changes in both lines are calculated as the difference between experimental and control beta values. For iMEF line, day 20 data was used. (**D**) Bar plot comparison of methylation difference correlations between SDHC-loss iMEFs, SDHB-loss imCCs, and SDHx-loss human PPGL. For iMEFs and imCCs, comparison is generated as difference between experimental and control beta values. iMEF comparison is generated at day 20 post induction of Sdhc rearrangement with doxycycline. For human PGL, comparison is difference of beta values between SDHx tumors and all others. (**E**) Venn diagram showing the overlap of gene promoters identified as hypermethylated (beta difference >0.05) as a consequence of iMEF SDHC loss, imCC SDHB loss, and human PPGL SDHx loss.

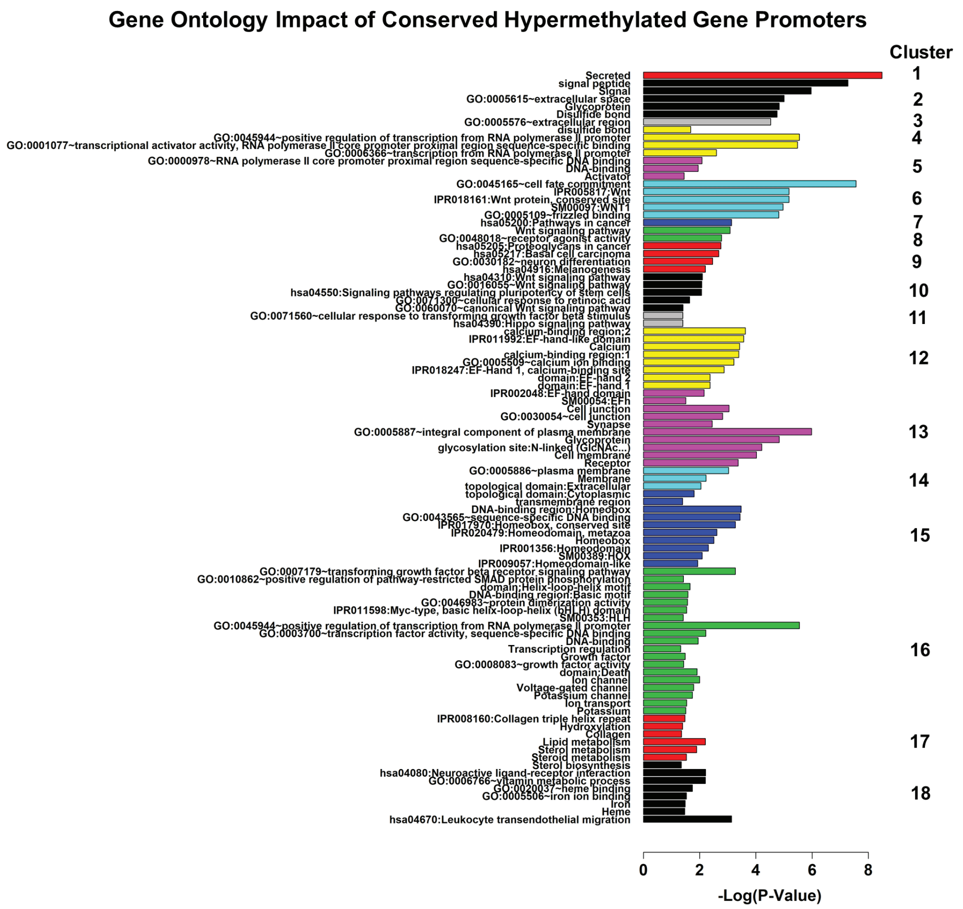

Supplementary Figure 3: DAVID functional enrichment analysis of gene ontologies identified as hypermethylated in Sdhc-loss iMEFs, SDHB-loss imCCs, and SDHx-loss human PPGL. Coloring corresponds to functional annotation cluster numbering.

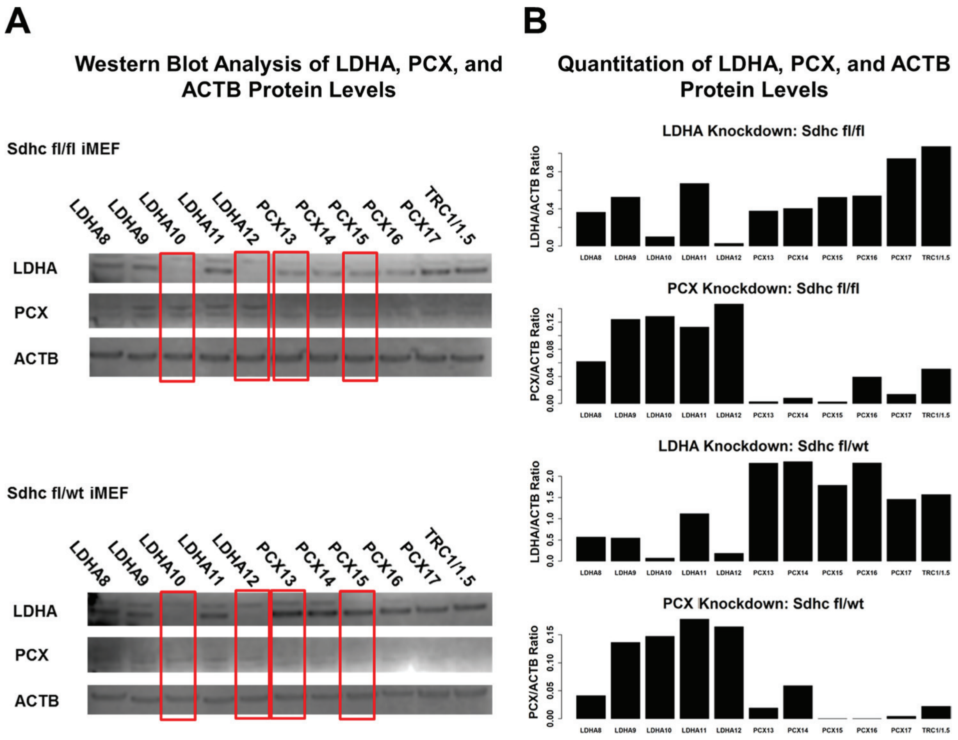

Supplementary Figure 4: Western blot analysis of stable shRNA-expressing lentivirus knockdown of LDHA and PCX in iMEFs. (**A**) Western blot analysis of LDHA, PCX, and ACTB protein levels in shRNA-expressing lentivirus knockdown lines. Five different shRNAs for each target are shown, as well as TRC1/1.5 non-targeted control. (**B**) Quantitation of LDHA and PCX protein levels, normalized to ACTB loading control.
